# Primary biliary cirrhosis associated with myasthenia gravis after postpartum: a case report

**DOI:** 10.1186/s13256-021-03092-x

**Published:** 2021-10-10

**Authors:** Lulu Zhang, Dongxue Ding, Liqiang Yu, Huan Qi, Chunru Han, Jianhua Jiang, Juean Jiang

**Affiliations:** 1 Department of Neurology, First Affiliated Hospital of Soochow University, Suzhou, 215031 China; 2 Department of General Medicine, First Affiliated Hospital of Soochow University, Suzhou, 215031 China

**Keywords:** Myasthenia gravis, Primary biliary cirrhosis, Postpartum

## Abstract

**Background:**

Autoimmune diseases refers to a class of diseases involving abnormal immune response of human body and tissue damage caused by the dysregulation of autoimmune balance or destruction of immune tolerance. Recent research has revealed that the occurrence of autoimmune diseases is influenced by genetic, hormonal, immunological, and environmental factors. As sex hormone levels change obviously during pregnancy and postpartum, the morbidity and recurrence rate of autoimmune diseases increase during this period.

**Case presentation:**

A 31-year-old Asian woman was admitted to our hospital for myasthenia gravis and treated with methylprednisolone and pyridostigmine bromide 3 months postpartum. Physical examination and laboratory inspection after admission suggested that the patient had primary biliary cirrhosis. Subsequently, azathioprine was added to the treatment, and the symptoms of both diseases were successfully controlled.

**Conclusions:**

This case exhibits a rare condition of myasthenia gravis combined with primary biliary cirrhosis postpartum. Given the fluctuation of the immune status during the postpartum period, combined autoimmune diseases need to be taken into account when patients develop clinical symptoms of an autoimmune disease. Therefore, detailed physical and laboratory examination can help to prevent the missed diagnosis of these diseases.

## Background

Myasthenia gravis (MG) is an acquired autoimmune disease mainly mediated by acetylcholine receptor antibody and primarily involving the acetylcholine receptor of the neuromuscular junction postsynaptic membrane. The specific hormone status during pregnancy and postpartum periods has a great effect on autoimmune diseases, potentially causing aggravation or recurrence of autoimmune diseases [1]. Studies suggest that the risk of developing MG is significantly higher in postpartum women than in non-postpartum women [2]. Moreover, the clinical manifestations of MG are more severe in women with pregnancy or postpartum [3].

Primary biliary cirrhosis (PBC) is a chronic intrahepatic cholestatic hepatopathy disease usually accompanied with clinical symptoms such as fatigue and itchy skin, and most commonly seen in middle-aged and elderly women. Progressive, nonsuppurative, destructive intrahepatic cholangitis is its pathologic feature and eventually leads to cirrhosis. The pathogenesis of PBC is not entirely understood, but it may be associated with abnormal autoimmunity caused by genetic background, environmental factors, and infection. Studies have shown that changes in sex hormones in women over time can increase the risk of PBC or worsen symptoms [4].

MG and PBC are two distinct autoimmune disorders, and it is rare for a patient to have both MG and PBC. Here we report a female patient who developed typical symptoms of MG after delivery, such as limb weakness that improved after rest, and with atypical symptoms of PBC. Upon review of the literature, the patient was diagnosed as having MG with PBC.

## Case presentation

A 31-year-old Asian woman presented with slurred speech and limb weakness. She first noticed slurred speech in September and subsequently developed drooping of the bilateral eyelid and, eventually, limb weakness. The above symptoms increased during periods of activity and improved after periods of rest. She was treated with pyridostigmine bromide (60 mg/8 hours) in a local hospital initially. The symptoms improved after taking medicine, but occasional weakness of both legs and chest tightness remained. Regarding past medical history, this patient underwent a cesarean section to give birth to her second child 3 months ago.

General physical examination revealed a yellow discoloration of facial skin and sclera. Neurological examination revealed mild dysarthria, mild droop of both side ptosis, and generalized muscle weakness after exercise. Weakness was most pronounced in the lower extremities. The upper eyelid fatigue test score was 2. Repetitive nerve stimulation showed that the amplitude of right axillary nerve decreased significantly at 1, 3, and 5 Hz, with a amplitude reduction of 35.6%, 45.2%, and 62.8% respectively. Besides, the amplitude of left facial nerve decreased by 13.9% and 17.5% at 3 Hz and 5 Hz. Reduced density of liver parenchyma is observed on contrast-enhanced chest computerized tomography (CT), and fatty liver may have been present. Positive results of laboratory tests are presented in Table 1. Rheumatoid factors, anti-nuclear antibody, anti-smooth muscle antibody and anti-thyroid antibody were negative. Thyroid hormone levels were within normal intervals. According to the clinical, electromyographic, and laboratory findings, the diagnosis of MG was unambiguous. In addition, the patient’s liver enzymes were elevated with positive AMA-M2 serology; according to the guidelines [5], she was diagnosed with PBC.

**Table 1:** Positive results of laboratory tests

Item	Result	Normal range
ESR (mm/hour)	34.00	0–26
AchR-IgG (U/mL)	12.10	< 0.45
TBIL (μmol/L)	44	3.4–17.1
DBIL (μmol/L)	14	0–6.8
IBIL (μmol/L)	30	1.7–10.2
ALT (U/L)	115.4	7–40
AST (U/L)	68.8	13–35
AMA-M2	Positive (++)	Negative

*ESR* erythrocyte sedimentation rate, *AchR-IgG* anti-acetylcholine receptor immunoglobulin G, *TBIL* total bilirubin, *DBIL* direct bilirubin, *IBIL* indirect bilirubin, *ALT* alanine transaminase, *AST* aspartate transferase, *AMA-M2* anti-mitochondrial antibody M2

The patient was diagnosed with MG after she came to our hospital. Therefore, she was treated with pyridostigmine bromide (60 mg/8 hours) and methylprednisolone after admission. The initial dose of methylprednisolone was 30 mg/day, then gradually increased to 80 mg/day for maintenance 3 days, and finally decreased to 30 mg/day for maintenance treatment. Ursodeoxycholic acid (250 mg/8 hours) and azathioprine (50 mg/12 hours) treatment was added after diagnosis of PBC. The patient’s condition improved steadily during 1 month of hospitalization. She continued to receive a combined treatment with pyridostigmine bromide (60 mg/8 hours), methylprednisolone (16 mg/12 hours), ursodeoxycholic acid (250 mg/8 hours), and azathioprine (50 mg/12 hours) after discharge.

During the 2 years follow-up after discharge, the patient’s methylprednisolone was gradually reduced to discontinuation within 1 year. Daily ursodeoxycholic therapy was administered strictly according to our instructions for 6 months after discharge, and the patient ceased ursodeoxycholic on her own. Interestingly, her liver enzyme indexes were all normal, although she stopped taking ursodeoxycholic acid after discharge. Now, the patient is receiving long-term maintenance treatment with pyridostigmine bromide (60 mg/8 hours) and azathioprine (50 mg/day). During the 2 years follow-up, the patient had no obvious discomfort except weight gain, and the relevant liver enzyme indexes were normal.

## Discussion and conclusions

This report firstly described the occurrence of MG and PBC in a patient in the postpartum period. In previous reports, the use of d-penicillamine in patients with PBC resulted in the development of MG [6]. However, patients can also develop both PBC and MG without d-penicillamine [7, 8]. In addition, it has been reported that patients could develop other autoimmune diseases (AID) in addition to PBC and MG [9]. According to previous reports, PBC combined with MG tends to occur more frequently in middle-aged and elderly women than men of the same age. This suggested that women can be more susceptible to autoimmune diseases in some conditions.

Studies have suggested that more than 80% of AID occur in women of childbearing age [10, 11], and AID is prone to relapse or aggravation during pregnancy and postpartum and improves after menopause [12]. The above phenomenon further confirmed that hormonal changes play an essential role in the occurrence and development of AID. The risk of AID is significantly increased in the first postpartum year and then decreased gradually due to the changes of sex hormones during pregnancy and postpartum [13, 14]. Serum levels of estradiol, progesterone, prolactin, and cortisol increase gradually during pregnancy. Estrogen and progesterone drop suddenly after childbirth, but prolactin levels remain high, especially in breastfeeding mothers [1]. Moreover, breastfeeding further reduces estrogen and cortisol levels [15, 16]. Changes in hormone levels lead to a strong immune tolerance during pregnancy, while the immune environment shifts to a proinflammatory state at the end of pregnancy and postpartum. The volatility of the immune state leads to bodies’ susceptibility to AID during this period.

This report plays a reference role in the diagnosis and treatment of AID in postpartum patients. Given the changes in immune status, it should be determined whether some other autoimmune diseases were complicated in the postpartum patients diagnosed with AID. Careful physical and laboratory examination can help to prevent the missed diagnosis of these diseases.

## Data Availability

The datasets used and/or analyzed during the current study are available from the corresponding author on reasonable request

## Abbreviations

AID: Autoimmune diseases
MG: Myasthenia gravis
PBC: Primary biliary cirrhosis
CT: Computed tomography
AMA: Anti-mitochondrial antibodies
ESR: Erythrocyte sedimentation rate
AchR-IgG: Anti-acetylcholine receptor immunoglobulin G
TBIL: Total bilirubin
DBIL: Direct bilirubin
IBIL: Indirect bilirubin
ALT: Alanine transaminase
AST: Aspartate transferase
AMA-M2: Anti-mitochondrial antibody M2
